# Homeostasis: The Underappreciated and Far Too Often Ignored Central Organizing Principle of Physiology

**DOI:** 10.3389/fphys.2020.00200

**Published:** 2020-03-10

**Authors:** George E. Billman

**Affiliations:** Department of Physiology and Cell Biology, The Ohio State University, Columbus, OH, United States

**Keywords:** physiology, homeostasis, internal milieu, Claude Bernard, Walter Cannon, control theory, feedback regulation—negative and positive, cybernetics

## Abstract

The grand challenge to physiology, as was first described in an essay published in the inaugural issue of Frontiers in Physiology in 2010, remains to integrate function from molecules to intact organisms. In order to make sense of the vast volume of information derived from, and increasingly dependent upon, reductionist approaches, a greater emphasis must be placed on the traditional integrated and more holistic approaches developed by the scientists who gave birth to physiology as an intellectual discipline. Our understanding of physiological regulation has evolved over time from the Greek idea of body humors, through Claude Bernard’s “milieu intérieur,” to Walter Cannon’s formulation of the concept of “homeostasis” and the application of control theory (feedback and feedforward regulation) to explain how a constant internal environment is achieved. Homeostasis has become the central unifying concept of physiology and is defined as a self-regulating process by which an organism can maintain internal stability while adjusting to changing external conditions. Homeostasis is not static and unvarying; it is a dynamic process that can change internal conditions as required to survive external challenges. It is also important to note that homeostatic regulation is not merely the product of a single negative feedback cycle but reflects the complex interaction of multiple feedback systems that can be modified by higher control centers. This hierarchical control and feedback redundancy results in a finer level of control and a greater flexibility that enables the organism to adapt to changing environmental conditions. The health and vitality of the organism can be said to be the end result of homeostatic regulation. An understanding of normal physiology is not possible without an appreciation of this concept. Conversely, it follows that disruption of homeostatic mechanisms is what leads to disease, and effective therapy must be directed toward re-establishing these homeostatic conditions. Therefore, it is the purpose of this essay to describe the evolution of our understanding of homeostasis and the role of physiological regulation and dysregulation in health and disease.

## Introduction

In November 2009, I agreed to launch a new open-access physiology journal to be called Frontiers in Physiology and the articles were published in April 2010. One of my duties as Field Chief Editor was to write a brief “Grand Challenge” article in which I discussed what I perceived to be the biggest challenges facing physiology as a discipline. As it has been 10 years since the publication of this first essay, it is an opportune time to re-visit and update this grand challenge article.

## The Grand Challenge in Physiology

In my 2010 essay, I stated that the grand challenge of physiology was “to integrate function from molecules to man” (Billman, 2010). In other words, to make sense of the vast volume of information derived from, and increasingly dependent upon, reductionist approaches. This, in my opinion, remains the most serious unmet challenge facing physiology today. A greater emphasis must be placed on the traditional integrated and more holistic approaches developed by the scientists who gave birth to physiology as an intellectual discipline. In other words, it time for physiologists to return our roots. It is no more possible to appreciate the beauty of de Vinci’s “Mona Lisa” or Van Gogh’s “The Starry Night” by removing and analyzing each individual dab of paint than we can understand how the various organ systems work together to maintain health by examining single genes or molecules. Just as when viewing a painting, the body can only be fully appreciated in its entirety. This essay will focus on the concept of homeostasis as the central organizing principle upon which the discipline of physiology is built, the very concept we need to return to in order to integrate function from molecule to the intact organism. Portions of the following sections were previously published in a slightly different form (Billman, 2013) and are reprinted with permission of the publisher.

## Homeostasis: a Definition

Homeostasis, as currently defined, is a self-regulating process by which biological systems maintain stability while adjusting to changing external conditions. This concept explains how an organism can maintain more or less constant internal conditions that allow it to adapt and to survive in the face of a changing and often hostile external environment. Our awareness of homeostasis has slowly emerged over the centuries and has become the central organizing tenet of physiology. If one does not understand this self-regulating process, then it is not possible to comprehend fully the function of the body in health and in disease. The disruption of homeostatic mechanisms is what leads to disease, and effective therapy must be directed toward re-establishing these homeostatic conditions, working with rather than against nature. In the following sections, the evolution of our understanding of homeostasis will be described and the role of physiological regulation and dysregulation in health and disease will be evaluated.

## Homeostasis: a Historical Perspective

“*True stability results when presumed order and presumed disorder are in balance. A truly stable system expects the unexpected, is prepared to be disrupted, waits to be transformed.*”

Tom Robbins (American Novelist, b. 1936)^1^

The concept that bodily regulation is required for health can be traced back to the ancient Greeks. The Greek physician/philosopher Alcmaeon of Croton (fl. 500 BC) proposed what can be called a “balance of opposites” to explain health and disease. He used a political analogy to define health and disease stating that: “*Health is the equality of rights of the functions, wet-dry, cold-hot, bitter-sweet and the rest; but single rule of either pair is deleterious.*” (Freeman, 1948). Thus, inequality of power leads to tyranny in a political system and disease in the body. This concept was expanded by Hippocrates of Kos (ca. 460–ca. 377 BC) who proposed that health was the product of the balance and mixture of four body fluids or humors: blood, phlegm, yellow bile, and black bile. He wrote that:

“*Health is primarily that state in which these constituent substances are in correct proportion to each other, both in strength and quantity and are well mixed. Pain occurs when one of these substances presents either a deficiency or excess, or is separated in the body and not mixed with the others.*” (Chadwick and Mann, 1950)

Thus, medicine became a process “*of subtraction and addition: subtraction of what is in excess, addition of what is wanting.*” (Jones, 1923). Hippocrates further recognized the role of nature’s helping hand in the healing process (*vis medicatrix naturae*), the ability of the body to heal itself (Hall, 1975). It was the role of the physician to clear the path so that nature could take its course. This concept became the basis for medicine in the ensuing centuries up to the dawn of the modern era.

Implicit in this concept of the “healing power of nature” is the assumption that the subunits of the body act in a cooperative manner to restore health when the normal state of the organism has been disturbed. Physiology, as a discipline dedicated to understanding how the parts of the body work together to maintain health, has its origins in the 16th century. The term physiology was first introduced by Jean Francois Fernel (ca. 1497–1558, Figure 1) in 1542 [*De Naturali Parte Medicinae* (on the natural part of medicine)] as the study of the function of the healthy body as distinguished from pathology, the study of disease (Hall, 1975). William Harvey (1578–1657) was the first individual to use carefully designed human and animal experiments to establish the function of a major bodily organ system with his description of the circulation of the blood. This application of physiology is illustrated in the following brief quotation from his seminal publication “*Exercitatio Anatomica De Motu Cordis et De Circulatione Sanguinis in Animalibus*” 1628 (Anatomical exercises on the motion of the heart and the circulation of blood in living creatures, first English translation 1653):

**FIGURE 1:**
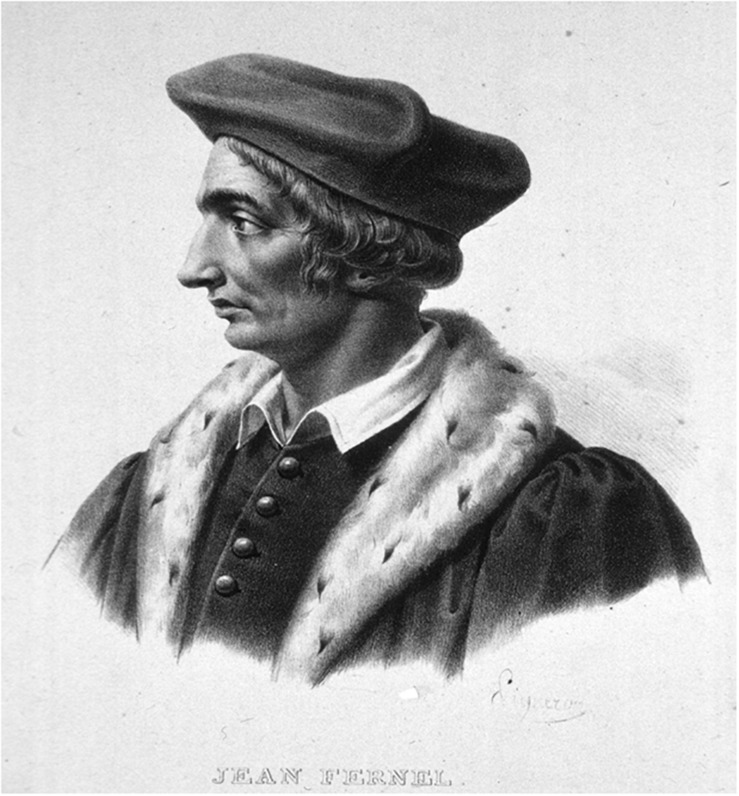
Portrait of Jean Fernel (ca. 1497–1558). He is the individual who coined the term physiology. Source: National Library of Medicine (the history of medicine public domain image files).

“It has been shown by reason and experiment that blood by the beat of the ventricles flows through the lungs and is pumped to the whole body … the blood in the animal body moves around in a circle continuously, and … the action or function of the heart is to accomplish this pumping. This is the only reason for the motion and beat of the heart.” (Harvey, 1628/1653)

Over the ensuing centuries, the concept of physiology has evolved, and a central tenet has emerged that unites the various sub-disciplines of physiology: the quest to understand how the various components of the organism work together to maintain a healthy state. It is only by understanding normal bodily function that the disruptions that lead to disease can be determined and ultimately corrected so as to restore the healthy state.

**FIGURE 2:**
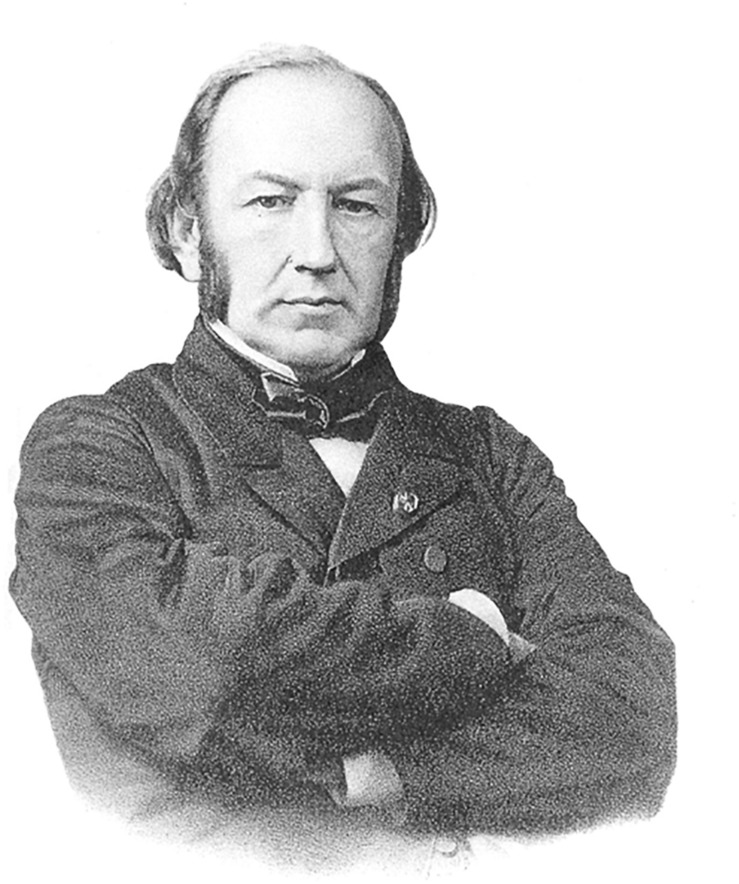
Photograph of Claude Bernard (1813–1878). He developed the concept of “*a fixité du milieu intérieur*,” that is, organisms maintain a stable internal environment despite changing external conditions. Source: National Library of Medicine (the history of medicine public domain image files).

**FIGURE 3:**
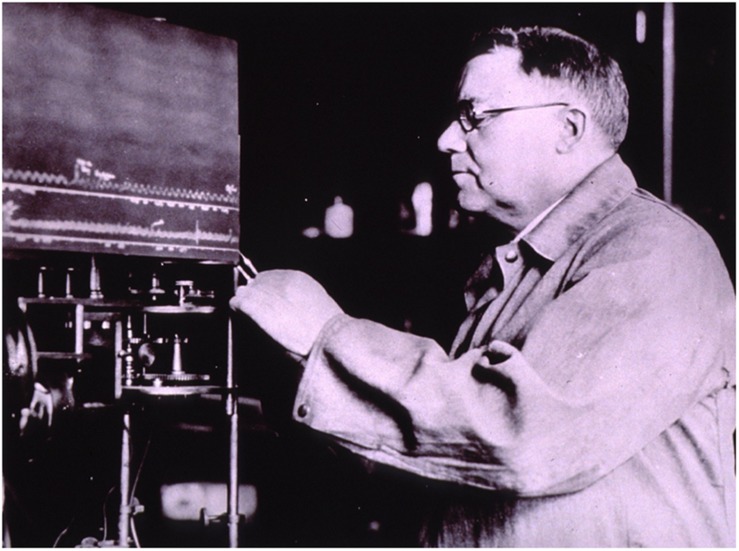
Photograph of Walter B. Cannon (1871–1945). He built upon the work of Claude Bernard and coined the word homeostasis to describe a self-regulating process by which biological systems maintain stability while adjusting to changing conditions. Source: National Library of Medicine (the history of medicine public domain image files).

As we have seen, a rudimentary understanding of the regulation and control of bodily function can be traced back to 6th century BC Greece. Despite sporadic progress over the centuries (Adolph, 1961), it was not until the 19th century that systematic physiological investigation produced major advancements on this concept. Our modern understanding of physiological regulation rests firmly on the shoulders of two giants in the field: Claude Bernard (Figure 2) and Walter Cannon (Figure 3) who described regulations in terms of the constancy of the internal environment and homeostasis, respectively.

The French Physiologist, Claude Bernard (1813–1878), who is often referred to as the founder of modern experimental physiology, was perhaps the first to appreciate fully that living systems possess an internal stability that buffers and protects the organism against a constantly changing external environment (Cooper, 2008). He recognized that the body possesses mechanisms that operate in a coordinated fashion to maintain a relatively constant temperature and blood glucose concentration and this internal stability was vital for the health of the organism. He concluded that: “*La fixité du milieu intérieur est la condition de la vie libre, independante*” (Bernard, 1865) [The fixity (i.e., constancy or stability) of the internal environment is the condition for the free, independent life]. What is often overlooked and needs to be stressed is that in this statement Bernard was proposing a new and radical hypothesis: the stability of the “*milieu intérieur*” was the antecedent to (i.e., required for) and not the consequence (outcome) of a free and independent life (Turner, 2017).

Although Bernard was highly honored and was the most famous French scientist during his lifetime, his hypothesis that the stability of the internal environment was independent of the external conditions, first articulated in 1854, was largely ignored for the next 50 years. Gross (2009) has proposed three reasons to explain the delay between the publication of Bernard’s ideas and their acceptance: (1) Pasteur’s exciting discoveries in bacteriology that had immediate application in the prevention and treatment of disease came to dominate biological investigations; (2) the gap between evolutionary thought and general physiology—it took time to appreciate that natural selection provided the means by which regulatory control could evolve; and (3) the technology necessary to measure the internal environment was not yet available.

However, by the late 19th century and early 20th century several investigators embraced Bernard’s ideas, both as a central explanatory concept and as a program for research in physiology. Among those influenced by Bernard were such physiological luminaries as William M. Bayliss, Ernest H. Starling, Joseph Barcroft, J. S. Haldane, and C. S. Sherrington in England, and L. J. Henderson and Walter B. Cannon in America (Adolph, 1961; Cooper, 2008; Gross, 2009). Starling, in fact, coined the phrase “the wisdom of the body” to describe the maintenance of a constant internal environment (Cooper, 2008). Walter Cannon later popularized this phrase when he used it as the title for his book in which he introduced the concept of homeostasis. In 1900, Charles R. Richet (1850–1935), a student of Bernard who later won the Nobel Prize in Physiology and Medicine, stressed the dynamic stability of the internal environment. The following quote, we shall see, presaged the definition supplied by Walter Cannon.

“*The living system is stable*…*it must be in order not to be destroyed, dissolved or disintegrated by colossal forces, often adverse, which surround it. By an apparent contradiction, it maintains its stability only if it is excitable and capable of modifying itself according to external stimuli and adjusting its response to the stimulation. In a sense, it is stable because it is modifiable – the slight instability is the necessary condition for the true stability of the organism.*” (Richet, 1900)

This concept of a constant internal environment (*milieu intérieur*) was expanded by the American Physiologist, Walter Cannon (1871–1945) (Cooper, 2008). He coined the term homeostasis from the Greek words Ǒμoιoς (hómoios) “similar” and στάσις (stásis) “standing still” (together to mean staying similar and *not* staying the same) to describe the self-regulating processes by which a biological system maintains stability while adjusting to changing environmental conditions. Homeostasis is often mistakenly taken to mean unchanging or stagnant. However, Cannon purposely selected the Greek word for similar, “hómoios,” rather than the word for same, “homo,” to express the idea that internal conditions could vary; that is, they are similar but not identical (stability but within range of values that allows the organism the freedom to adapt). Homeostasis, then, is the tendency of a system to maintain an internal stability as the result of the coordinated response of its parts to any situation or stimulus that disturbs normal conditions or function. Thus, the term homeostasis attempts to convey two ideas: (1) an internal stability within a range of values and (2) the coordinated dynamic response that maintains this internal stability (self-regulatory goal-seeking behavior). As he explained in the following quote from his highly influential monograph, “The Wisdom of the Body,” published in 1932:

“*The coordinated physiological processes which maintain most of the steady states in the organisms are so complex and peculiar to living beings – involving, as they may, the brain and nerves, the heart, lung, kidneys and spleen, all working cooperatively – that I have suggested a special designation for these states, homeostasis. The word does not imply, something set and immobile, a stagnation. It means a condition – a condition which may vary, but is relatively constant.*” (Cannon, 1963)

As emphasized by Cannon, homeostasis is not static; it is, rather, a dynamic self-adjusting system that maintains viability in the face of changing environmental demands. Echoing Bernard, homeostasis is a unique property of living organisms and, may be responsible for life itself. More recently, Turner (2017) described homeostasis as a dynamic disequilibrium – dynamic, as a stable internal environment requires continuous monitoring and adjustments (once again, a self-regulatory process) in order to maintain a balance between opposing forces (what he calls disequilibrium) so that a free and independent life is possible. He went further and stated that “*properly understood, homeostasis is life’s fundamental property, what distinguishes it from non-life. In short, homeostasis is life.*” (Turner, 2017).

The final piece of the homeostasis puzzle was supplied by the application of control theory from systems engineering to explain self-regulation in biological systems. The “constancy” of internal physiochemical conditions is then largely maintained by the often complex interaction of multiple negative (and positive) feedback systems. The interaction of these regulatory mechanisms not only increases the stability of the system but provides redundancy (back-up) such that failure of one component does not necessarily lead to catastrophe. Thus, from its inception physiological investigations have been directed toward understanding the organism (be it microbe, plant, animal, or human) as a *single functional entity*.

## Feedback Regulation: the Process That Underlies Homeostasis

“*Nam deteriores omnes sumus licentiate.*” (*We all degenerate in the absence of control*)

Terence (Heauton Timorumenos, line 483)

As we have seen, a critical feature of homeostasis is that an organism’s internal environment is held within a narrow range of values via a self-adjusting (a goal-seeking) system. Both feedback and feedforward are the mechanisms by which homeostasis is obtained. I shall begin this section with a discussion of the contribution of feedback to homeostatic regulation and then briefly discuss feedforward (also known as central command) mechanisms.

A feedback system is a closed loop structure in which the results of past actions (changes in the internal environment) of the system are fed into the system (via information, feedback) to control future action; the system affects its own behavior (modified from Forrester, 1976). There are two types of feedback systems: negative feedback that seeks a goal and responds as a consequence of failure to meet this goal (maintains a stable *range* of values) and positive feedback that produces growth processes wherein the actions build on the results that then generate still greater action (a growth cycle). These feedback systems are themselves subject to higher levels of control; that is, the operational range of the regulated variables can be adjusted to support the behavioral response to environmental stimuli. Homeostasis is the result of the complex interaction and competition between multiple negative and positive feedback systems and provides the basis for physiological regulation.

Once again we can trace the origin of self-regulatory systems to the ancient Greeks.

The first documented device that employed the principle of self-regulation was a water clock (clepsydra) invented by Ktesibios (or Ctesibius, Greek Kτησίβις) of Alexandria (fl. 285-222 BC) (Landels, 2000). A water clock depends upon a steady flow of water to measure an unvarying flow of time. If the water level is not relatively constant, the water outflow will vary depending on the height of the water column supplying the clock (faster with a full container and slower as the water level in the container falls). The water clock designed by Ktesibios used a float valve (similar to that used in the modern flush toilet) to maintain a constant water level in the clock water reservoir. As water levels fall, the float also falls thereby opening a valve that allows water to flow into the clock reservoir and to replenish the water level. Then, as the water returns to the desired level, the float rises and closes the valve. Thus, the clock water reservoir could be regulated such that there is no net gain or loss in the water level and thereby it maintains a constant water outflow rate from which an accurate estimate of time can be obtained. The accuracy of this type of water clock was not supplanted until the 17th century when a pendulum was employed to regulate the clock mechanism.

**FIGURE 4:**
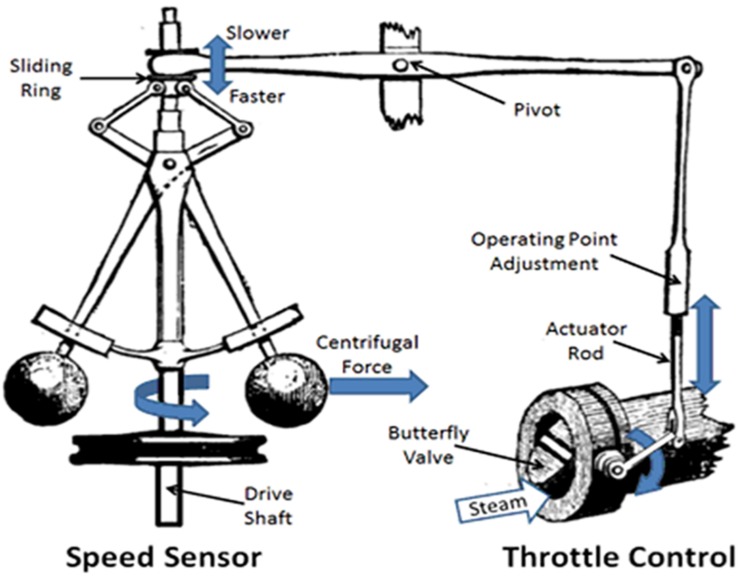
Schematic representation of James Watt’s steam engineer flyweight governor. See text for details. Source: public domain, as modified from, https:www.mpoweruk.com/figs/watt_flyball_governor.htm.

A number of other self-regulatory devices were invented in the ancient and medieval periods but it was not until the late 18th century, with the invention of the steam engine that the study of devices that incorporated “corrective feedback” for regulation became a subject for systematic investigation. A major limitation of early steam engines was that their speed was affected by both the steam pressure generated by the boiler and work load placed upon the engine. James Watt (1736–1819) vastly improved the efficiency and safety of the steam engine by the development of a centrifugal feedback valve that controlled the speed of the engine (Rosen, 2010). This “governor” (Figure 4) employed a pair of metal balls spinning on each side of a rotating vertical shaft aligned in such a manner that as the engine speed increased so also did the spinning rate of metal balls (called flyweights) and, as a consequence of increased centrifugal force, the balls would spread apart. This, in turn, opened a valve to decrease the flow of steam into the engine and a slower speed was restored. Conversely, as the engine speed decreased, so also would the rotation of the flyweights, thereby decreasing the outward centrifugal force. The flyweights would drop (pulled down by gravity) closer together, closing the steam valve so more steam could enter into the engine and increase its speed. As with the water clock and its water reservoir level, a constant engine speed could be maintained despite fluctuating steam pressure and changing work load without the constant supervision of a human monitor.

Later in the 19th century, James Clerk Maxwell (1831–1879) published a mathematical analysis of Watt’s governor that established the principles for understanding self-regulating devices and became the foundation upon which control theory is built (Maxwell, 1868). In 1927, Harold S. Black (1898–1983) applied feedback regulation to electrical circuits to amplify transatlantic telephone signals (Black, 1934). His negative feedback amplifier (patented in 1937) can be considered to be one of the most important developments in the field of electronics. Further advances in systems control theory were achieved during World War II with the development of servo-control (negative feedback) mechanisms for anti-aircraft weapons.

In 1943, two influential papers were published that established that the mathematical principles of control theory, as first described by Maxwell, could be applied to explain behavior in living organisms. Arturo Rosenblueth, Norbert Wiener, and Julian Bigelow’s paper entitled “Behavior, Purpose and Teleology” (Rosenblueth et al., 1943) and Warren McCulloch and Walter Pitts’, “A Logical Calculus of the Ideas Immanent in the Nervous Activity” (McCulloch and Pitts, 1943) were the first to establish a link between the self-regulating nature of physiological processes in living animals and negative-feedback systems designed by engineers. Interestingly, Rosenblueth worked closely with Cannon and undoubtedly was influenced by his ideas. A few years later, Wiener (1894–1964) introduced the term cybernetics [from kybernetes (κυβερνήτης), the Greek word for governor (as in steersman or pilot)] to describe the study of self-regulatory control and communication in the animals (Wiener, 1961). In his book Cybernetics, Wiener (1961) developed the first formal mathematical analysis of feedback control in biological systems, concepts that have subsequently been extensively applied in modeling physiological systems as, for example, by Arthur Guyton (1919–2003) and his many students with regard to cardiovascular regulation. Thus, the concept of feedback regulation in living organisms may be said to have co-evolved with the mathematical concepts of control theory in mechanical systems. Negative feedback regulation is a particularly important mechanism by which homeostasis is achieved, as will be described in the following paragraphs.

The water clock and centrifugal steam governor described in the preceding paragraphs provide classic examples of negative feedback systems. As we have seen for the water clock, the opening and closing of the float/valve creates a cycle where information about the water level can be fed back into the system to effect changes to maintain the water level at some constant pre-determined value. Thus, the float simultaneously affects the water levels and is affected by water level forming a circular causality or a cycle of causation. It is important to emphasize that this is an automatic self-regulatory system, meaning that it requires no external adjustment once the operating level around which the variable is regulated has been set.

**FIGURE 5:**
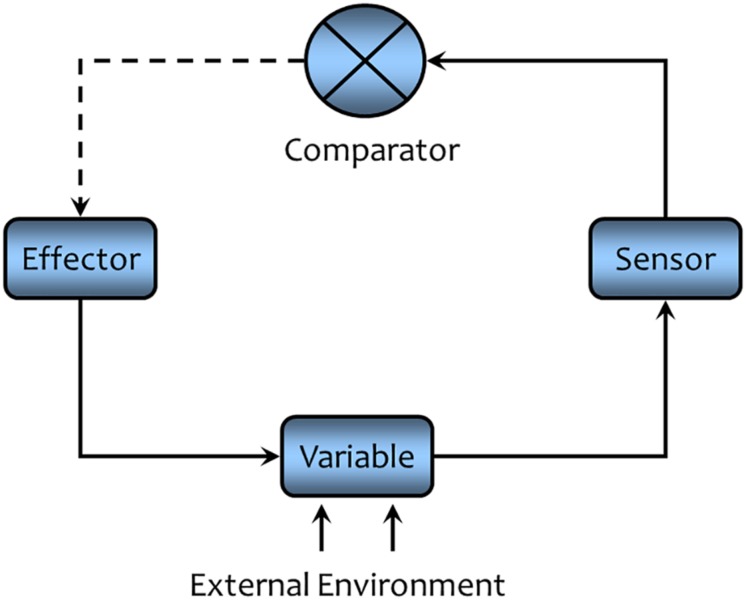
A schematic representation of negative feedback regulation. A solid line indicates that the connected components are directly related (an increase in one component leads to increase the connected component, while a decrease will lead to decrease in the connected components). A dashed line indicates the connected components are inversely related (an increase in one component leads to a decrease in the connected component while a decrease will lead to an increase in the connected component). An odd number of dashed lines are a necessary condition for any negative feedback cycle of causation. Negative feedback acts to maintain the controlled variable within a narrow range of values (see text for a detailed description).

A simplified general form of a closed loop feedback system is illustrated in Figure 5. The illustrated cycle consist of four main components, (1) the variable (or set of variables) that are to be controlled, (2) a sensor that monitors the variable of interest, (3) a comparator or central processing unit (mathematically, the transfer function—the input/output relationship) where the information provided by the sensor (afferent or sensory pathway) is fed back into the system. The information is compared with the “desired” state (set point or operating point) to detect any error (difference between the desired state and the prevailing state), and (4) effectors (efferent or motor pathways) that are activated to correct any error. Effector activity opposes and thereby buffers against changes in the variable. A solid line is used in this diagram to indicate a direct relationship (increase leads to increase, decrease leads to decrease) between the components, while a dashed line represents an inverse relationship (increase leads to a decrease and vice versa). Negative feedback regulation must contain an odd number of dashed lines in order to maintain the variable within a narrow range of the desired value.

**FIGURE 6:**
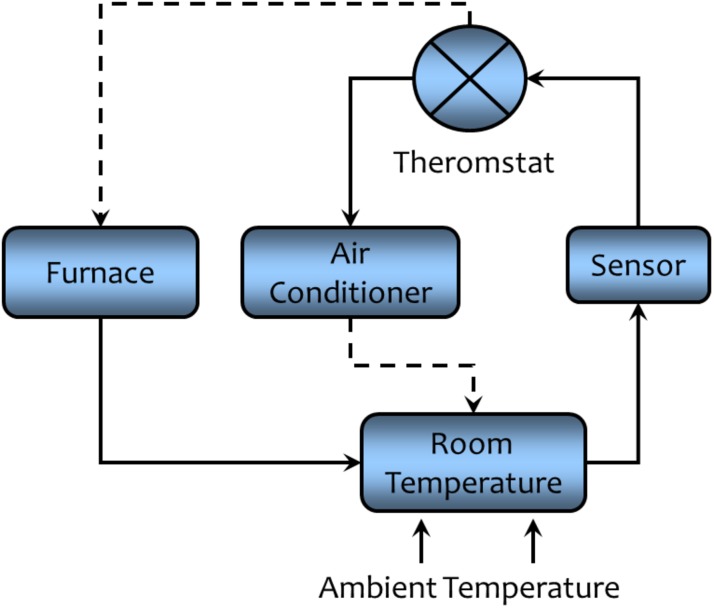
A schematic representation of the regulation of room temperature to illustrate the concept of negative feedback regulation. A solid line indicates that the connected components are directly related (an increase in one component leads to an increase the connected components, while a decrease will lead to a decrease in the connected components). A dashed line indicates that the connected components are inversely related (an increase in one component leads to a decrease in the connected component while a decrease will lead to an increase in the connected component). Negative feedback acts to maintain the room temperature within a narrow range of values despite changes in ambient temperature (see text for a detailed description).

A commonly used example of negative feedback is the regulation of room temperature by a thermostatically controlled heating and cooling system as displayed in Figure 6. Room temperature is the regulated variable, the sensor is a thermometer, the comparator is the thermostat—the device that compares the desired temperature (operating point) with the actual temperature (error detection), and the effector is the heating or cooling system. In this example, an increase in outside heat is detected by the sensor and the information is conveyed to the thermostat. The temperature information is compared to operating point and if there is sufficient difference between actual and desired temperature, the cooling system is activated and the heating system is inactivated (reducing the error signal). The converse would happen if environmental temperature should fall, the cooling system would be turned off and the heating units activated. Thus, stable room temperatures can be maintained despite a wide range of fluctuating external conditions.

**FIGURE 7:**
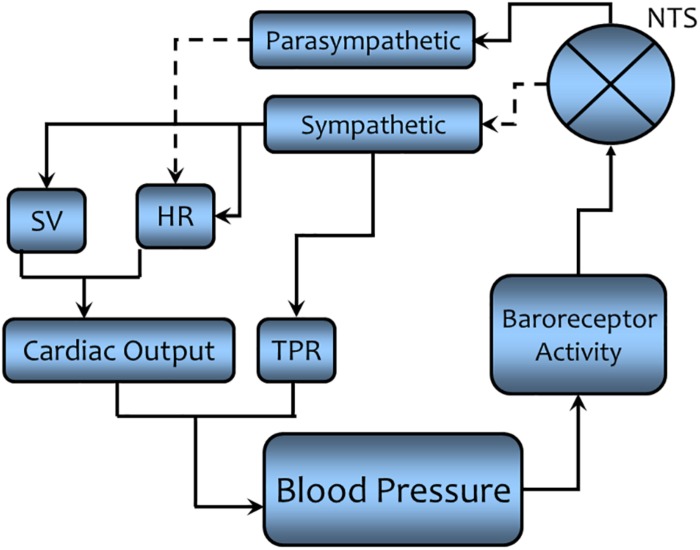
A simplified schematic representation of the regulation of arterial blood pressure as a physiological example of negative feedback regulation. A solid line indicates that the connected components are directly related (an increase in one component leads to an increase the connected components, while a decrease will lead to a decrease in the connected components). A dashed line indicates the connected components are inversely related (an increase in one component leads to a decrease in the connected component while a decrease will lead to an increase in the connected component). Negative feedback regulation acts to maintain the arterial blood pressure within a narrow range of values (see text for a detailed description). NTS = nucleus tractus solitarius, the site where sensory information is processed and the efferent response is initiated. It acts as a “barostat” analogous to the “thermostat” in room temperature regulation. SV = stroke volume (the amount of blood ejected by the heart with each ventricular contraction), HR = heart rate, the number of beats (ventricular contractions) per minute, TPR = total peripheral resistance, the resistance to the forward movement of blood (inversely related to the blood vessel diameter).

It must be emphasized that feedback regulation in biological systems (living organisms) is much more complex than the simple “clockwork” feedback systems described in the preceding paragraphs for mechanical systems. With this caveat firmly in mind, the concept of self-regulation in biological system can be illustrated by the regulation of blood pressure. As early as the mid-19th century, it became obvious that arterial blood pressure was maintained within a narrow range of values via the activation of neutrally mediated reflex adjustments (Adolph, 1961). However, it was not until to 1960s that the principles of negative feedback were applied to explain the homeostatic regulation of arterial blood pressure. A detailed description of intricacies of blood pressure regulation is beyond the scope of the present essay (for a recent review see Dampney, 2016). Nonetheless, a *simplified* feedback cycle, analogous to the one we used for room temperature, is seen in Figure 7.

Before we can discuss this figure, we first must mathematically define arterial pressure using Ohm’s law expression (for a hydraulic rather than for an electrical circuit). Algebraically, blood pressure (BP – analogous to voltage, E, in an electrical circuit) is the product of the cardiac output (CO – analogous to current, I, in an electrical circuit) and systemic vascular resistance also known as total peripheral resistance (TPR – analogous to electrical resistance, R). Cardiac output is itself the product of the amount of blood ejected per beat [stroke volume (SV)] multiplied by the number of beats per minute [heart rate (HR)].

So that, BP = SV × HR × TPR. (E = I × R for an electrical circuit).

**FIGURE 8:**
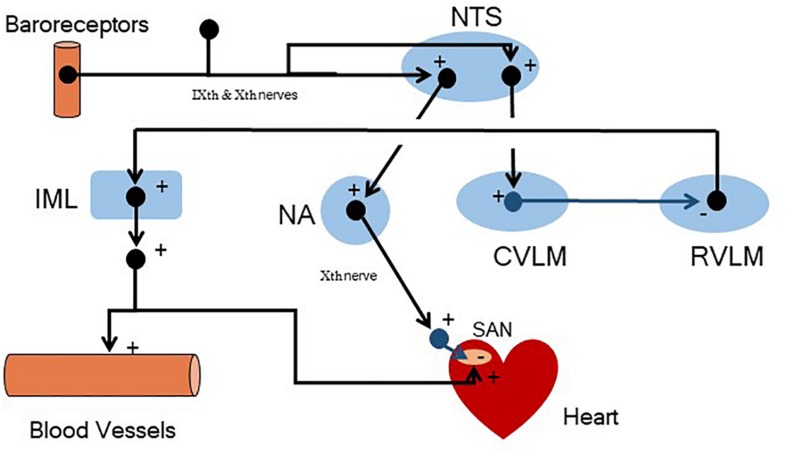
A simplified schematic representation of the central neural structures involved in baroreceptor reflex regulation of arterial blood pressure. Arterial pressure receptors located in the carotid sinuses and aortic arch (nerve firing increases as arterial pressure increases) convey afferent information via the glossopharyngeal (IXth) and vagus (Xth) nerves to the brain, respectively. This information is first processed by neurons located in the nucleus tractus solitarius (NTS). The NTS then alters parasympathetic and sympathetic efferent nerve activity. Specifically, the NTS alters the activity of neurons (monosynaptically) located in the nucleus ambiguus (NA, parasympathetic pre-ganglionic neurons) and neurons (polysynaptically, via interneuron connections) in the caudal ventrolateral medulla (CVLM). The CVLM, in turn, regulates the tonic sympathetic activity that originates in the rostral ventrolateral medulla [RVLM, that regulates sympathetic pre-ganglionic neurons located in the intermediolateral column (IML) of the spinal cord]. + = excitatory neurotransmitters (shown in black); – = inhibitory neurotransmitters (shown in blue); SAN = sino-atrial node. As an example, an increase in arterial blood pressure would increase baroreceptor nerve firing, increasing NTS neuron activity which, via interneurons, would trigger both an increase in the activity of the parasympathetic pre-ganglionic neurons located in the NA and decrease the firing of sympathetic pre-ganglionic neurons located in the IML (less directly via CVLM mediated inhibition of the tonic activity of the RVLM). The net result would be a decrease in heart rate (? cardiac parasympathetic and↓ cardiac sympathetic nerve activity), stroke volume (↓ cardiac sympathetic nerve activity), and arteriolar vasoconstriction (↓ total peripheral resistance, ↓ cardiac sympathetic nerve activity). Reductions in arterial blood pressure would provoke changes in the opposite direction. Note that the sign changes at the heart (parasympathetic effects on the SAN) and within the medulla (CVLM mediated inhibition of the RVLM). This “sign change” is necessary for negative feedback regulation.

It is evident that changes in arterial blood pressure can be countered by corrective changes in either the output from the heart (SV and/or HR) or resistance to movement of blood through blood vessel (by adjusting vessel diameter, diameter is inversely related to TPR) or both. Returning to Figure 7, the sensors are receptors (baroreceptors) located in arterial blood vessels (aortic arch and carotid sinuses) that respond to changes in arterial pressure (increases in BP increase receptor activity). The comparator function is performed by a cluster of nerve cells within the medulla of brain [nucleus tractus solitarius (NTS)] where the signal is processed to affect the output of the effector system. It acts as a “barostat” a function analogous to the thermostat in the regulation of room temperature shown in Figure 6. The signal is processed at the NTS and then effects excitatory [rostral ventral lateral medulla (RVLM) via interneuron connections] and inhibitory [nucleus ambiguus (NA), monosynaptically] areas within the medulla to elicit the motor response (see Figure 8 for more details). The motor output from the central nervous system to target organs is conducted by means of two sets of nerves to the heart: parasympathetic nerves (originating in the NA) that decrease HR and sympathetic nerves (originating in the intermediolateral column, IML of the spinal cord, regulated by neurons from the RVLM) that increase HR and SV. The sympathetic nerves also go to blood vessels, the activation of which decreases vessel diameter and thereby increases TPR. Thus, if BP should increase, the so-called baroreceptor reflex is activated. An increase in parasympathetic activity coupled with a decrease in sympathetic activity would reduce cardiac output (decreasing HR and SV) and decrease TPR. The opposite changes would occur if blood pressure should decrease. Thus, negative feedback regulation buffers against transitory changes and thereby helps maintain a stable blood pressure on a beat-by-beat basis throughout the day despite changing environmental or behavioral conditions.

Feedforward regulation is another mechanism by which homeostasis is modified and maintained as part of the behavioral response to environmental stimuli. During feedforward regulation, which is also often referred to as central command, a response is elicited without feedback about the status of the regulated variable; that is, disturbances are evaluated and adjustments are made *before* changes in the regulated variable have actually occurred. For example, returning to constant room temperature, feedforward regulation would entail activation of the furnace as soon a window or door is opened during a cold winter day before the thermostat detects a change in the ambient temperature, In a similar manner, blood pressure, cardiac output, and skeletal muscle blood increase in anticipation of fighting or fleeing a potential danger (the defense reaction) or when an athlete envisions running the race before the starter’s pistol has been fired (see below). It should be emphasized that feedforward regulation, while acting independently of changes in the regulated variable, does require information about the nature and extent of the potential disturbance. For room temperature, the status of the windows and doors (whether they are open or not) must be monitored (sensors placed on these openings). Otherwise, a response would not be elicited until room temperature had deviated sufficiently from the set point to be detected by the thermostat (and thereby activate the previously described negative feedback response). In living organisms, learning and experience provide the information necessary for feedforward control. A cat soon learns the difference between a mouse (food) and the neighbor’s dog (a dangerous and barking nuisance) and will react accordingly (making the appropriate behavioral and physiological adjustments for appetitive or aversive stimuli).

**FIGURE 9:**
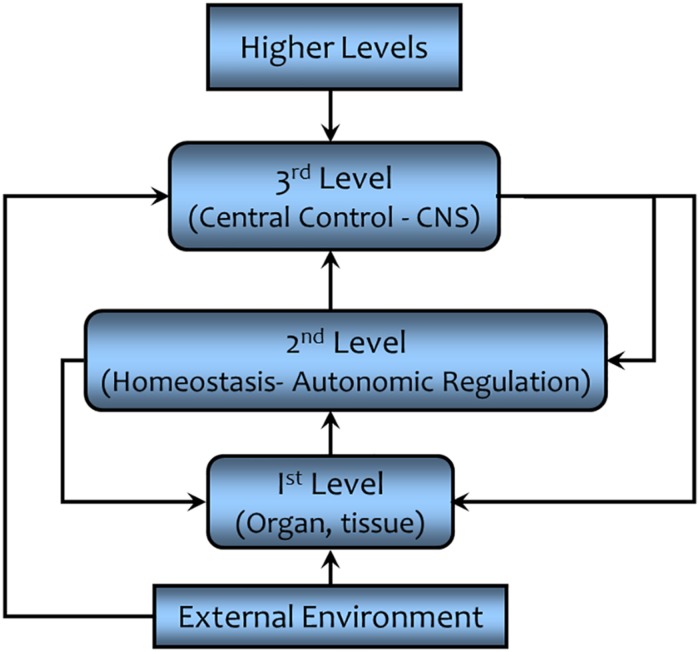
A simplified schematic representation of the higher order control of homeostatic regulation. This hierarchical control results in a finer level of control and a greater flexibility that enables the organism to adapt to changing environmental conditions (see text for details). CNS = central nervous system.

The simple negative feedback schema described in the preceding paragraph cannot adequately convey the complexity of the homeostatic process that allows an organism to function and adapt to changing environmental conditions (Carpenter, 2004). For example, the operating point (or more accurately the operating range) of the negative feedback regulation can be adjusted or even overridden by higher levels of control (Goodman, 1980). These adjustments of the automatic (e.g., feedback) regulation allow the organism to adapt and to respond appropriately to changing external conditions. This hierarchical control is a multi-level, multi-goal seeking system as shown in Figure 9 (modified from Goodman, 1980). In this schematic diagram, the first level represents the physiochemical processes, the organ and tissue functions, the component parts upon which homeostasis acts. The second level is autonomous (self) regulation, homeostasis (e.g., baroreceptor reflex). Here changes in a given variable are sensed and adjustments of the first level processes are initiated without input from higher levels of control. The third level is found in the central command and control centers (central nervous system) that process the information transmitted from the second level and integrates it with information from other sensory inputs to coordinate the physiological and behavioral response to changing environmental conditions. The higher centers can “intervene,” making the adjustments as required to support the autonomic (i.e., autonomous and automatic) processes. This control can occur either at the conscious or unconscious level. An example of a conscious intervention would be the initiation of behaviors to cope with changing room temperature – adding or removing clothing, opening or closing windows seeking shade or sun, etc. – while an example of subconscious control would be the adjustments in blood pressure regulation during exercise (a shift in the operating point of the baroreceptor reflex so that both HR and SV increase despite increases in BP as compared to resting conditions; Raven et al., 2006). Thus, the third level coordinates behavioral and physiological responses to the external environment in order to maintain comfort and to ensure survival. However, it must be emphasized that higher level control is not possible if the first level components do not function properly. Finally, one could also envision even higher levels of control, factors outside of the organism.

The “autopilot” in a modern jet airliner can be used to illustrate the levels of control (Wiener, 1961). Once the preferred heading, attitude, and airspeed have been set, the autopilot will maintain level flight within acceptable degrees of roll, pitch, and yaw, despite changes in wind speed or minor turbulence. However, take-off and landing (at least until “self-driving” technology has been perfected) require the direct intervention of the human pilot. Thus, the first level consists of the components of the airliner, the jet engines, and the airframe (fuselage, wings, flaps, rudder, etc.), the second level is the autopilot, and third level is the human pilot. In this example, a fourth level of control of the airplane is exerted by the air traffic controllers who provide directions to the pilot while an even higher level of control would reside in the Federal Aviation Administration (FAA) that sets the policy followed by the air traffic controllers.

The cardiorespiratory response to exercise provides a physiological example of this hierarchical control of homeostatic regulation. The first level consists of the tissues and organs that form the cardiovascular and respiratory system (heart, lung, and blood vessels, but also the kidneys and endocrine glands that regulate salt and water retention and thereby blood volume), the second level of control is the baroreceptor (direct effect) and cardiorenal reflexes (indirect via regulation of blood volume), the third level of regulation takes place within the medulla (NTS) of the central nervous system where the sensory information is processed and the efferent response initiated. The medullary structures are themselves regulated by higher centers (e.g., hypothalamus and motor centers) in the brain. In fact, the hypothalamus plays a major role in coordinating (matching) changes in the internal environment with the behavioral response to external challenges. As previously mentioned, HR and BP are simultaneously elevated during exercise demonstrating that baroreceptor reflex regulation has been altered. These adjustments are required in order to increase oxygen delivery so that it can match the increased metabolic demand of the exercising muscles. Raven et al. (2006) have demonstrated that these adjustments result from shifting the baroreceptor reflex to a higher operating point (i.e., altering the range of homeostatic regulation) rather than from an inhibition of this reflex. Both feedback (sensory information for the exercise muscle, the so-called exercise pressor reflex) and feedforward (central command: for example, anticipation of the onset of exercise, such as visualizing the race before it is run, will increase HR, BP, and skeletal muscle blood flow) contribute to these reflex adjustments. Finally, higher levels of control include the starter who determines when the race will begin, the event organizers who determine what races are run, and the sports regulatory agencies (Olympic committee, FIFA, NCAA, etc.) that set the rules that govern the event.

Homeostatic control of the internal environment, therefore, involves much more than simple negative feedback regulation (Carpenter, 2004). The hierarchical levels of command and control allow the organism to adjust its internal conditions to respond, to adapt, and to meet the challenges placed upon it by a changing and often hostile environment. Adaptation can, in fact, be viewed as an emergent property of homeostasis and may be responsible for the life’s unique nature (Turner, 2017).

## Homeostasis: Implication for Reductionism

“…*All the kings’ horses and all the kings’ men*

*Could not put Humpty Dumpty together again*”

Traditional English Nursery Rhythm (earliest published version 1803)

(Opie and Opie, 1997)

The concept of homeostasis has important implications with regard to how best to understand physiology in intact organisms. In recent years, reductionist (attempts to explain the nature of complex phenomena by reducing them to a set of ever smaller and simpler components; the view that the whole is merely the sum of its parts), rather than holistic approaches have become dominant, not only in physiology, but in science in general. The earliest glimmerings of reductionist thought can be found in the surviving fragmentary writings of Thales and other pre-Socratic Greek philosophers who speculated that all matter was composed of various combinations of four key elements: earth, air, fire, and water (the four humors of the body correspond to these elements) (Hall, 1975). The pinnacle of Greek reductionism is found in the work of Leucippus and his student Democritus who proposed that all things consist of an infinitely large number of indivisibly small particles that they called atoms (Hall, 1975). The modern application of reductionism in science can be traced to Francis Bacon (1561–1620) and Rene Descartes (1596–1650). Bacon incorporated reductionism as a central component, along with inductive reasoning, in his new empirical method (*Novum Organum 1620, as* opposed to Aristotle’s *Organon* a treatise on logic and syllogism, i.e., deductive reasoning) (Bacon, 1620) for the attainment of knowledge in natural philosophy, what has subsequently become known as the scientific method. Descartes likewise embraced reductionism as the pathway to knowledge, albeit with an emphasis on deduction (rationalism) rather than induction (empiricism) as advocated by Bacon. In his “Discourse on the Method of Rightly Conducting One’s Reason and Seeking Truth in Science,” Descartes (1637) introduced two concepts that would have profound impact on biological investigations. In this, his most influential treatise, he described four precepts to arrive at knowledge. The second and third precepts, in particular, exemplify the reductionist’s approach as follows:

“*The second to divide each of the difficulties under examination into as many parts as possible and as might be necessary for its adequate solution*”

“*The third to conduct my thoughts in such order that, beginning with those objects that are simplest and most readily understood, I ascend little by little, and as it were, step by step, to the knowledge of the more complex.*” (Descartes, 1637)

His second and more far reaching conclusion was that the body was merely a machine. Thus, it was assumed that by applying Cartesian reductionism, one could deduce the complex physiology of the intact organism by understanding the presumably simpler functions of the individual organs and their constituent parts (from the molecular level to subcellular organelles to cells to tissue to organ and finally back to the intact organism).

There can be no denying the power of this approach. In only a few decades after DNA was identified as the molecule of inheritance, its sequence of the some 3 billion base pairs has been mapped for humans and other species, the genetic “code” for protein synthesis has been broken, and between 20,000 and 25,000 human genes that regulate a multitude of proteins have been determined. Humpty Dumpty quite literally has been smashed into a billion pieces.

However, reductionism rests upon the unstated assumption that the parts somehow entail the whole, that complexity is merely the product of incomplete understanding. In other words, the assumption that once we have gathered enough information (big data) and have developed sufficient computing power (ultra-fast computers), we can put Humpty back together again. The salient question is then whether this assumption is correct? Although we have sequenced the genome for many species, we have little understanding of the process by which the genome becomes an organism. We now know, in intricate detail, the basis for neuronal action potentials and synaptic transmission but do not understand how these electrical and chemical events give rise to consciousness. Complexity may not be the illusion it once naïvely was thought to be. As elegantly described by Claude Bernard more than 150 years ago:

“*Physiologist and physicians must never forget that a living being is an organism with its own individuality. Since physicists and chemists cannot take their stand outside the universe, they study bodies and phenomena in themselves and separately, without necessarily having to connect them with nature as whole. But physiologists, finding themselves, on the contrary, outside the animal organism which they see as a whole, must take account of the harmony of the whole, even while trying to get inside, so as to understand the mechanism of its every part. The result is that physicists and chemists can reject all idea of the final causes for the facts that they observe; while physiologists are inclined to acknowledge a harmonious and pre-established unity in an organized body, all of whose partial actions are interdependent and mutually generative. We really must learn, then, that if we break up a living organism by isolating its different parts, it is only for the sake of ease in experimental analysis, and by no means in order to conceive them separately. Indeed, when we wish to ascribe to a physiological quality its value and true significance, we must always refer to this whole, and draw conclusions only to its effects in the whole.*” (Emphasis added, Bernard, 1865)

It cannot be overstated that the whole *is* greater than the sum of the parts!

The grand challenge faced by contemporary physiology in this post-genomic era as first described in 2010 (Billman, 2010) remains how to integrate and to translate this deluge of information obtained *in vitro* into a coherent understanding of function *in vivo*. Although a machine may consist of many parts, the parts in isolation do not make the machine. Anyone who has tried to assemble a child’s bicycle on Christmas Eve can testify that the parts do not a machine make. In an analogous fashion, while organisms are made of molecules, molecules are not organisms. The concept of one gene, one protein, one function is woefully inadequate to explain the dazzling complexity and startling beauty of the living organism – the intricate dance of homeostatic mechanisms necessary for a “free and independent life.” A sequence of base pairs in the DNA molecule can no more explain the complexities of life than a series of 1s and 0s on a compact disc recording can explain the emotional response to music (Noble, 2006). Man and other organisms are not mere vehicles for the perpetuation of genes, selfish or otherwise. The days for reductionist deconstruction are numbered; more holistic and integrated systems approaches are required to put Humpty Dumpty back together again. It is time for physiologist to return to their roots and consider the organism as a whole as advocated by Claude Bernard.

A second, and by no means less important, challenge will be to train the next generation of scholars to perform the integrative studies in intact preparations (whole animals or organs) that are the pre-requisite for clinical applications. Unfortunately, there has been a progressive decline in the number of integrative physiology training programs, resulting in a paucity of individuals with the skill sets necessary for whole animal *in vivo* experimentation. The problem is exacerbated by the renaming or actual elimination of Departments of Physiology within Colleges of Medicine. It currently is fashionable for physiology departments to rechristen themselves as “Departments of Molecular Biology/Physiology.” With tongue firmly in cheek, one wonders if Departments of Atomic Physiology will be soon in the offing.

With the increasing emphasis on molecular and genetic approaches, it is not unusual to find members of physiology departments who have not even taken an introductory course in physiology. This is, indeed, a shame as much of the excitement for physiology as an intellectual discipline can best be encountered in the student lab. Nothing can replace the hands-on learning nor instill a better appreciation for the concept of homeostasis than performing these classic physiology experiments. In the student lab, one can go beyond the dry textbook description of physiological principles and see them in action. The student can experience, first hand, the same excitement and sense of wonder that the earlier investigators must have had when they first examined skeletal muscle-nerve function in frogs, saw the clearance of dye in the easily visible glomeruli in the necturus (mudpuppy), or pondered the mysteries of cardiopulmonary regulation in mammals (rat, rabbit or dogs). Thus, it very much remains an open question as to whether a sufficient number of suitably trained investigators will be available to meet the grand challenge: to integrate function from molecules to intact organisms.

## Summary

Our understanding of physiological regulation has evolved over time from the Greek idea concerning the balance between the body humors, through Claude Bernard’s “milieu intérieur” to Walter Cannon’s formulation of the concept of homeostasis and the application of control theory (feedback regulation) to explain how a constant internal environment is achieved. Homeostasis has become the central unifying concept of physiology and is defined as a self-regulating process by which a living organism can maintain internal stability while adjusting to changing external conditions. Homeostasis is not static and unvarying; it is a dynamic process that can change internal conditions as required to survive external challenges. This is made clear by the care Cannon used when coining the word homeostasis. He deliberately selected Greek words that when, combined, meant “staying similar” rather than “staying the same” to emphasize that internal conditions could vary yet still produce stability (within a range of values rather than a single value). Thus, homeostasis does not mean “stagnation.” It is also important to note that homeostatic regulation is not merely the product of a single negative feedback cycle but reflects the complex interaction of multiple feedback systems that can be modified by higher control centers. This hierarchical control and feedback redundancy produces both a finer level of control and a greater flexibility that enables the organism to adapt to changing environmental conditions. The health and vitality of the organism can be said to be the end result of homeostatic regulation of the internal environment; an understanding of normal physiology is not possible without an appreciation of this concept. Conversely, it follows that disruption of homeostatic mechanisms is what leads to disease, and effective therapy must be directed toward re-establishing these homeostatic conditions, working with rather than against nature.

## Author Contributions

GB prepared all aspects of this review article.

## Conflict of Interest

The authors declare that the research was conducted in the absence of any commercial or financial relationships that could be construed as a potential conflict of interest.

## Footnotes

1 http://www.brainyquote.com/quotes/quotes/t/tomrobbins404093.html
